# Adsorptive removal of crystal violet from aqueous solution by ultrasonic-assisted synthesized zirconium-2,6-naphthalenedicarboxylate metal-organic framework

**DOI:** 10.55730/1300-0527.3495

**Published:** 2022-08-23

**Authors:** Penmethsa Kiran KUMAR, Sunkara Satya VENI

**Affiliations:** 1 Department of Chemistry, Government Degree College, Chodavaram, Andhra Pradesh, India; 2 Department of Chemistry, Jawaharlal Nehru Technological University Kakinada, Kakinada, Andhra Pradesh, India

**Keywords:** Adsorption, crystal violet, metal-organic framework, ultrasound-assisted, zirconium

## Abstract

Zirconium-2,6-naphthalenedicarboxylate metal-organic framework (Zr-NDC MOF) was prepared using ultrasound-assisted synthesis and tested for the adsorptive removal of crystal violet (CV) dye from aqueous solution. The pristine Zr-NDC was characterized using powder X-ray diffraction, scanning electron microscopy, thermogravimetric analysis, and energy-dispersive X-ray spectroscopy, Fourier-transform infrared, elemental analysis, and dynamic light scattering techniques. The maximum percentage removal of CV dye was found to be 99.45% with an initial dye concentration of 10 mg L^−1^. The kinetics and adsorption isotherm models were used to investigate the removal of CV dye from aqueous solution using Zr-NDC. Langmuir adsorption isotherm model (R^2^ = 0.9880) describes the adsorption of CV dye onto Zr-NDC and the maximum equilibrium adsorption capacity (454.2 mg g^−1^) was achieved with the CV dye having an initial concentration of 100 mg L^−1^. The adsorption was found to follow pseudo-second-order kinetics (R^2^ = 0.9960) with a rate constant of 6.52 × 10^−4^ g mg^−1^ min^−1^. The effect of various parameters such as dye concentration, contact time, pH of dye solution, and MOF dose on the adsorption of dye was investigated. The study proved that the Zr-NDC is a promising adsorbent in the removal of CV dye from aqueous solution.

## 1. Introduction

Crystal violet (CV), also known as methyl violet 10B, is a synthetic basic cationic dye having antibacterial, antifungal, and anthelmintic properties. It is regarded as a biohazard substance and acts as a mitotic poison and a carcinogen. The presence of CV dye in water bodies results in undesirable colorations which, in turn, leads to low light penetrations for photosynthetic activities. Removal of CV from industrial wastewater is essential for the mitigation of environmental pollution and the safety of human and animal health [1–3].

Several physicochemical methods such as adsorption, photochemical degradation, precipitation, electrochemical degradation, ion exchange, and biological methods were reported to effectively remove dyes from industrial wastewater [4–7]. Among these, adsorption is a reliable method as it has several advantages like ease of operation, high efficiency, and low cost of application. Several adsorbents such as activated carbon, clay material, agricultural waste, and biomaterials were tested to remove dye from the aqueous solution effectively. MOFs perform better as adsorbents than conventional adsorbents in removing dyes from the aqueous solution. Structural diversity, porous structure, large surface area, and presence of active sites in the framework are some of the characteristics which make MOFs more advantageous for adsorptive removal of dyes from aqueous solution [8–11]. Furthermore, there are several challenges in employing bare MOFs for the removal of organic dyes using other methods, especially by photocatalytic degradation which makes adsorptive removal by MOFs an attractive choice [12,13]. Several metal-organic frameworks were used for the removal of CV dye [14–24]. So far, no study has been reported on the adsorptive removal of CV dye from aqueous solution by zirconium-based MOF.

Metal-organic frameworks (MOFs) are highly ordered porous crystalline materials. In recent years, zirconium-based MOFs have attracted the attention of the research community owing to their exceptionally high thermal, hydrothermal, mechanical, and chemical stability [25,26]. Based on Pearson’s hard/soft acid/base (HSAB) concept, the ultrahigh stability of zirconium-carboxylate MOFs can be attributed to the covalent character of Zr-O bonds [27]. Modulated synthesis strategy has been adopted in the synthesis of Zr-based MOFs since it enhances the reproducibility of the synthesis procedures and increases the crystallinity of MOFs [28].

Compared to various traditional and newly developed synthetic protocols, ultrasound-assisted synthesis for the preparation of MOFs is straightforward, convenient, and highly controllable [29]. Synthesis of MOFs performed under ultrasonic conditions is energy efficient and hence an environmentally friendly method [30].

Zr-NDC is one of the least studied members of the family of UiO-based MOF. It was synthesized using the solvothermal method by Zhang et al. in 2013 [31]. So far, four different Zirconium-2,6-naphthalenedicarboxylate MOFs namely, DUT-52(Zr) and DUT-84(Zr) by Kaskel group, MIL-140B by Serre group, and Zr_6_O_4_(OH)_4_(NDC)_6_.xDMF by Maître group have been reported in the literature [32–34]. Zr-NDC has exhibited promising applications as luminescent material for sensing small molecules, the electroluminescent active material in OLED devices, and catalytic electrodes for highly efficient solar light-driven photocatalytic disinfection [35–36]. There is only one published report concerning the ultrasonic-assisted synthesis of zirconium-based MOFs [37]. So far, the preparation of Zr-NDC using ultrasound-assisted synthesis was not reported in the literature.

In the present study, Zr-NDC was synthesized for the first time using ultrasonication. Modulated synthesis strategy was used in the synthesis of MOF. The efficacy of pristine Zr-NDC for the effective removal of CV dye from the aqueous solution was investigated. The effect of various parameters such as dye concentration, adsorbent dose, contact time, and pH of dye solution on the adsorption was studied to optimize the application of Zr-NDC as an adsorbent in the removal of CV dye from aqueous solution. The adsorption isotherms and kinetics of adsorption in the adsorptive removal of CV dye by Zr-NDC were also studied.

## 2. Experimental section

### 2.1. Materials and instrumentation

All the chemicals were obtained from Sigma Aldrich and were used without any further purification. Powder X-ray diffraction (PXRD) patterns were recorded using a Bruker D8 Advance diffractometer equipped with monochromatized Cu Kα radiation (λ = 1.5406 Å) operated at 40 kV and 40 mA. Scanning electron microscopy (SEM) images were taken on a JEOL JSM-6390LA instrument. The acceleration voltage was set to 20 kV. Elemental analysis via energy-dispersive X-ray spectroscopy (EDS) was performed using an acceleration voltage of 10 kV so that zirconium (Lα = 2.042 keV), carbon (kα = 0.277 keV) and oxygen (kα = 0.525 keV) could be qualitatively determined. The elemental analysis for carbon, hydrogen, and nitrogen was performed with an Elementar Vario EL III analyzer. The Fourier transform infrared (FT-IR) spectra were obtained on Agilent Cary 660 instrument working in the transmission mode in the 400–4000 cm^−1^ range. Thermogravimetric analysis (TGA) measurement was carried out using Perkin Elmer STA 6000 thermal analyzer. Dynamic light scattering (DLS) measurements were performed on a Zetasizer (Nano-ZS, Malvern) to determine the hydrodynamic parameters of the Zr-NDC MOF particles in water. Nitrogen adsorption-desorption isotherms were obtained on a BELSORP analyzer at −196 °C. A Shimadzu UV-2450 spectrophotometer was used to determine the absorbance of dye solutions in the visible region. An ultrasonic bath (USC 300, ANM) was used for ultrasonication. Ultrasonic irradiation having a frequency of 40 kHz with a power of 150 watts was used. To alter the pH of the dye solution, 0.1 M HCl and 0.1 M NaOH solutions were used. A digital pH meter (Model 335, Systronics) was used to measure the pH of the solutions.

### 2.2. Synthesis of Zr-NDC metal-organic framework

The synthesis of Zr-NDC is outlined in Scheme. Acetic acid was used as the modulator. 2,6-naphthalenedicarboxylic acid (320 mg, 1.5 mmol) was added to a solution of ZrOCl_2_.8H_2_O (480 mg, 1.5 mmol) in DMF (12 mL) and then acetic acid (2.7 mL, 45 mmol) was added. The resulting solution was placed in an ultrasonic bath and subjected to ultrasonic irradiation of 40 kHz (150 watts) for a period of 3 h. The temperature was monitored during the reaction and was observed to be less than 60 °C even after sonication for 3 h. After cooling down to room temperature, the as-synthesized white crystalline MOF was recovered by filtration. The solid material was washed three times with 10 mL of DMF followed by ethanol (3 × 10 mL) and acetone (3 × 10 mL). The material was dried overnight at room temperature and then activated by heating at 120 °C under a vacuum for 6 h. Under similar reaction conditions, crystalline Zr-NDC MOF was not obtained with the formic acid modulator. The attempts to obtain single crystals of MOF were also unsuccessful.

### 2.3 Adsorption study

Removal of CV dye from aqueous solution by adsorption was studied using as-synthesized Zr-NDC as the adsorbent. The adsorption studies were carried out using variable amounts of MOF, dye solutions having different initial concentrations, varying the pH of dye solution, and contact time of MOF with the dye solution. These studies were carried out at a temperature of 30 ± 2 °C. A standard solution of the dye was prepared by dissolving 1000 mg of the dye in 1000 mL of distilled water. A set of standard solutions was prepared using the stock solution. The absorbance maximum of dye solutions at 590 nm was measured and a calibration curve was plotted. In all the adsorption experiments, 100 mL of dye solution was used. The dye solution and MOF were mixed using a magnetic stirrer at 200 rpm. The adsorption efficiency,

q_e_ (mg g^−1^), was calculated using Equation 1.


Equation 1 $$q_{e}=\frac{V\;(C_{0}-C_{e})}{M}$$

where C_o_ is the initial dye concentration (mg L^−1^) and C_e_ is the dye concentration (mg L^−1^) at equilibrium. V is the volume of the dye solution (L) and M is the mass of Zr-NDC (g).

Twenty milligrams of Zr-NDC was added to each of the 100 mL dye solutions having a concentration of 10, 20, 30, 40, 50, 60, 70, 80, 90, and 100 mg L^−1^. After the time interval of 12 h, the absorbance of each solution was measured and the C_e_ values were determined from the absorbance values using the calibration curve.

The percentage removal of dye was calculated using Equation 2.


Equation 2 $$Percentage\;removal=\frac{\left(C_{o}-C_{e}\right)}{C_{o}}\times 100\%$$

Adsorption isotherms were analyzed using three different adsorption isotherm models: Freundlich, Langmuir, and Temkin [38–39].

Freundlich adsorption isotherm is expressed as Equation 3.


Equation 3 $$ln\;q_{e}=ln\;K_{F}+\frac{1}{n}\;ln\;C_{e}$$

Here K_F_ is the Freundlich constant and n is the constant whose value indicates how favorable the adsorption process is.

Langmuir isotherm is represented by Equation 4.


Equation 4 $$\frac{C_{e}}{q_{e}}=\frac{C_{e}}{q_{m}}+\frac{1}{q_{m}K_{L}}$$

Here q_m_ is the maximum adsorption capacity (mg/g) of the adsorbent (Zr-NDC) with the highest concentration of dye (100 mg L^−1^) used in the adsorption experiment and K_L_ is the Langmuir adsorption constant.

Temkin isotherm is given by Equation 5.


Equation 5 $$q_{e}=\frac{RT}{B_{T}}\;ln\;C_{e}+\frac{RT}{B_{T}}\;ln\;K_{T}$$

Here B_T_ is the adsorption heat constant (J) and K_T_ is the equilibrium binding constant (L mg^−1^).

### 2.4 Adsorption kinetics

For the kinetic study, 100 mL of dye solution having a concentration of 10 mg L^−1^ and 20 mg of Zr-NDC as adsorbent was used. At the desired time (t), the dye solutions were withdrawn and the absorbance was measured. The dye amounts adsorbed at time t, q_t_ (mg g^−1^) was calculated using Equation 6.


Equation 6 $$q_{t}=\frac{V\;(C_{0}-C_{t})}{M}$$

Here C_t_ is the dye concentration (mg L^−1^) at time t (min).

Three kinetic models, pseudo-first-order, pseudo-second-order, and intraparticle diffusion were applied to study the rate of removal of dye in the adsorption process [40]. The three models are expressed as Equations 7–9.

Pseudo-first-order kinetics,


Equation 7 $$ln(q_{e}-q_{t})=ln\;q_{e}+k_{1}t$$

Pseudo-second-order kinetics,


Equation 8 $$\frac{t}{q_{t}}=\frac{1}{k_{2}q_{e}^{2}}+\frac{1}{q_{e}}t$$

Intraparticle diffusion kinetics,


Equation 9 $$q_{t}=k_{i}t^{1/2}+C$$

Here k_1_ (min^−1^), k_2_ (g mg^−1^ min^−1^) and k_i_ (mg g^−1^ min^−1/2^) are the rate constants for pseudo-first-order, pseudo-second-order, and intraparticle diffusion respectively. C is the intraparticle diffusion constant.

## 3. Results and discussion

### 3.1. Characterization of Zr-NDC

**Figure 1:**
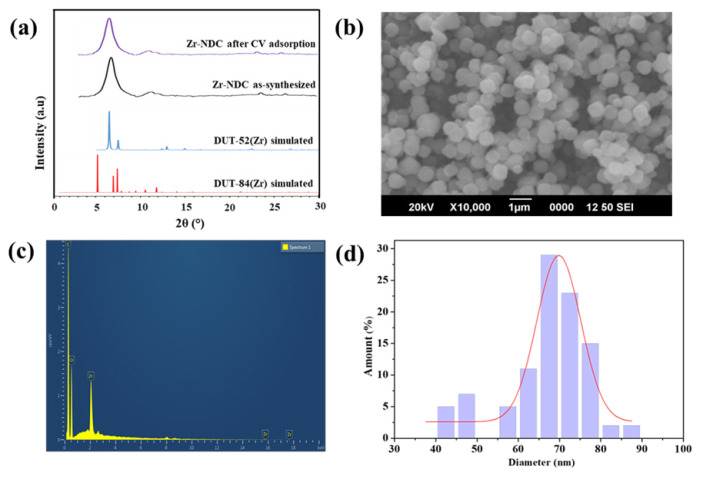
Characterization of Zr-NDC MOF with different methods (a) Comparison of the experimental PXRD patterns of Zr-NDC MOF with the simulated DUT-52(Zr) and DUT-84(Zr) and PXRD pattern after CV adsorption. (b) SEM image. (c) EDS spectrum. (d) Particle size distribution of Zr-NDC MOF particles from SEM images.

The pristine Zr-NDC was characterized using different characterization techniques. The MOF was highly crystalline and the crystals had spherical morphology with homogenous size distribution as evident from PXRD patterns and SEM images in Figures 1a and 1b. The sharp and high-intensity peak below 2θ of 10° in PXRD patterns indicated the formation of the Zr-NDC metal-organic framework [41]. The PXRD patterns of Zr-NDC did not exactly match with the simulated DUT-84(Zr), and DUT-52(Zr) patterns. Based on the comparison between simulated and as-synthesized PXRD patterns of DUT-84(Zr) and DUT-52(Zr) MOFs reported earlier, we confirmed the formation of Zr-NDC MOF. A comparison between PXRD patterns before and after adsorption by Zr-NDC indicates that the MOF framework was stable after the adsorption of CV dye. The energy-dispersive X-ray spectroscopy (EDS) result of Zr-NDC is given in Figure 1c. In the spectrum, peaks corresponding to carbon, oxygen, and zirconium were identified based on the energies of emitted x-rays from these elements. Using the SEM images, the diameters of nearly 50 Zr-NDC particles were measured with ImageJ. The obtained values were plotted in the form of a histogram fitted with Gaussian distribution as presented in Figure 1d. The average diameter of MOF particles was 67 nm with a standard deviation of 10 nm.

**Figure 2:**
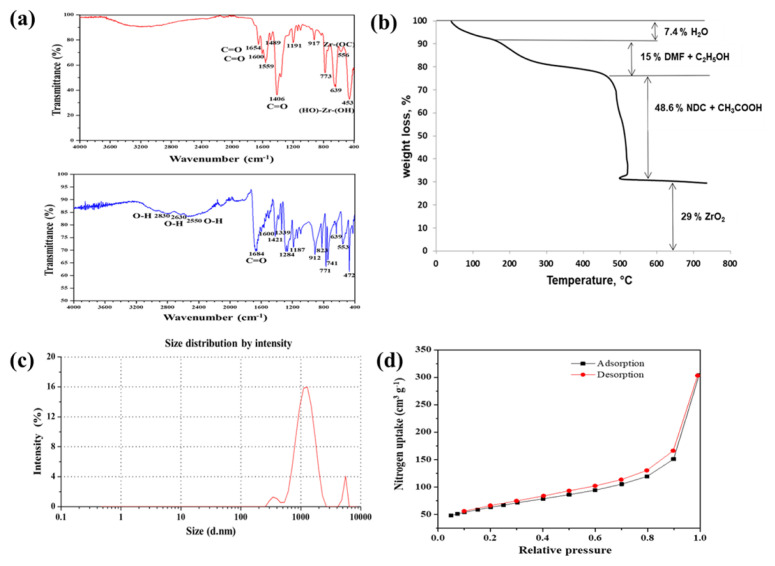
(a) FT-IR spectrum of Zr-NDC MOF (top) and 2,6-NDC (bottom). (b) TGA curve of Zr-NDC MOF. (c) Particle size distribution of Zr-NDC MOF from DLS. (d) N_2_ adsorption-desorption isotherms of Zr-NDC MOF at 77 K.

The FT-IR spectrum of Zr-NDC recorded in the region 400–4000 cm^−1^ was compared with that of 2,6-naphthalenedicarboxylic acid (free organic linker) in Figure 2a. There were significant differences observed among the two FT-IR spectra. The C=O stretching vibration observed in the free organic linker (1684 cm^−1^) was shifted to a lower wavenumber in the MOF spectrum, and two strong bands observed at 1406 and 1600 cm^−1^ can be attributed to symmetric and asymmetric stretching vibrations of carboxylate ions which were due to the reaction between organic linkers and the metal ions. The absorption bands in the region 2500–3000 cm^−1^ corresponding to the stretching vibrations of the O-H bonds in the free organic linker were no longer present in the MOF spectrum. The absorption band at 556 cm^−1^ and 453 cm^−1^ were ascribed to the asymmetric stretching vibration of Zr-(OC) and bending vibration of (OH)-Zr-(OH) bonds respectively. Based on the previous reports, the C=O stretching frequency observed at 1654 cm^−1^ corresponds to DMF. Since, in liquid DMF, this characteristic band was located around 1675 cm^−1^, the shifting of the absorption band to a lower wavenumber suggests that the DMF molecules were coordinated to the metal nodes of the Zr-NDC through the carbonyl groups [42,43].

Figure 2b shows the thermogravimetric data for the Zr-NDC. The TGA curve was similar to DUT-84(Zr) curve. Three distinct mass loss regions were observed in the thermogram. Mass loss from 40 to 140 °C could be attributed to the volatilization of adsorbed water. Further increase in the temperature leads to a weight loss of 15% due to the cleavage of coordinated DMF and ethanol molecules. The framework decomposition starts above 460 °C. Pure ZrO_2_ is formed as the final solid product due to the decomposition of the organic linker and acetic acid from the framework. The characteristic decomposition pattern observed in the TGA curve supports the composition of the synthesized Zr-NDC. From TGA analysis, it was evident that the pristine Zr-NDC was defective and there were missing organic linkers in the MOF structure. Acetic acid and DMF are coordinated to the metal as compensating ligands to compensate for the loss of coordination due to the missing organic linkers. FT-IR spectroscopy and elemental analysis data also support these facts.

Elemental analysis: Found, %: C, 38.13; H, 2.10; N, 1.42. Zr_6_O_4_(OH)_4_(NDC)_4_(CH_3_COO)(C_2_H_5_OH)_3_ (DMF)_2_. Calculated, %: C, 39.68; H, 3.36; N, 1.49.

To explore the solution-based applications of MOF particles such as drug delivery, Zr-NDC particles were characterized in solution with DLS [44]. The particle size distribution of Zr-NDC is shown in Figure 2c. Of Zr-NDC particles, 91.5% are distributed at 1230 nm with a polydispersity index (PDI) of 0.368. The bigger size of MOF particles in the solution state compared to the solid state can be attributed to the presence of aggregates of particles in the solution. Figure 2d illustrates the nitrogen adsorption-desorption isotherms of Zr-NDC. The BET surface area, pore volume, and average pore diameter were found to be 226.7 m^2^/g, 0.47 cm^3^/g, and 8.29 nm respectively.

### 3.2. Effect of initial dye concentration

**Figure 3:**
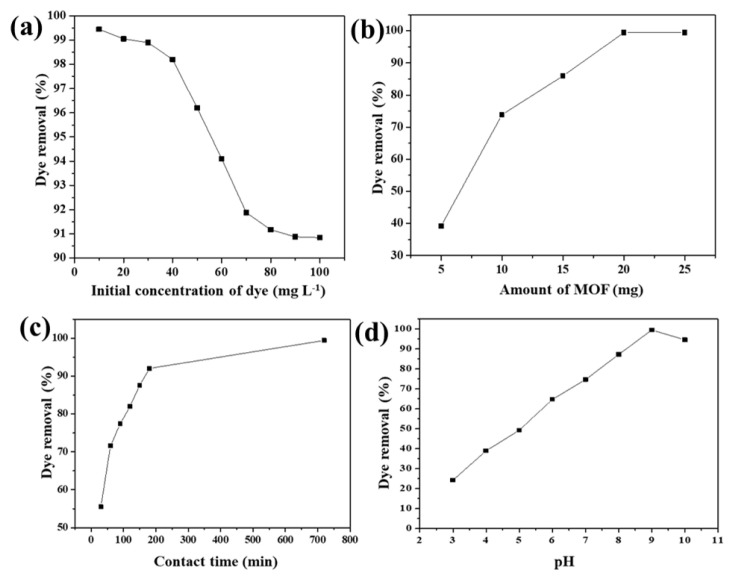
Factors affecting the adsorption of CV dye onto Zr-NDC MOF (a) Initial concentration of CV dye. (b) Amount of Zr-NDC MOF dose. (c) Contact time. (d) pH of the solution.

Using 20 mg of Zr-NDC adsorbent, the adsorption experiment was carried out by varying the initial concentration of CV dye in the range of 10–100 mg L^−1^. The equilibrium time for adsorption was 12 h. The effect of the initial concentration of dye on the adsorption by Zr-NDC was shown in Figure 3a. The percentage removal of dye decreased from 99.45% to 90.84% with the increase in the initial concentration of dye from 10 mg L^−1^ to 100 mg L^−1^. As reported in the earlier study, the decrease in the percentage removal of dye can be attributed to the saturation of adsorption sites with the initial dye concentration of 10 mg L^−1^ [14].

### 3.3. Effect of Zr-NDC dose

The effect of the Zr-NDC dose on the percentage removal of CV dye in the adsorption experiments was shown in Figure 3b. In the adsorption experiments, a Zr-NDC dose of 5,10,15,20, and 25 mg was added to 100 mL of 10 mg L^−1^ CV dye solution. The percentage removal of dye was increased from 39.21% to 99.45% with the increase in the dose of Zr-NDC from 5 mg to 20 mg due to the increase in surface area of the MOF. Maximum dye removal percentage was achieved with 20 mg of MOF dose.

### 3.4. Effect of contact time

The variation in the percentage of dye removal with the contact time was shown in Figure 3c. Initially, there was a sharp rise in the percentage of dye adsorbed onto the Zr-NDC surface with contact time. Afterward, there was a gradual slowdown in the percentage of dye adsorbed with time. With the increase in contact time, there will be a decrease in the number of active sites on the surface of Zr-NDC leading to a decrease in the extent of adsorption. The equilibrium time for adsorption was found to be 12 h. The maximum dye removal percentage achieved was found to be 99.45%.

### 3.5. Effect of pH

pH is one of the key factors affecting the efficiency of adsorbents in the removal of dye from the aqueous solution. Earlier studies revealed that variation in pH of the solution will alter the surface properties of the adsorbent and the degree of ionization of the absorptive molecules [45]. To study the effect of the pH of the solution, adsorption experiments were carried out with an initial CV dye concentration of 10 mg L^−1^ and Zr-NDC adsorbent dose of 20 mg at a temperature of 30 ± 2 °C for 12-h equilibrium time. The variation in percentage removal of dye by adsorbent with the change in the pH of the solution is shown in Figure 3d. There was a gradual increase in percentage removal with the increase in pH of the solution from 3.0 to 9.0. The dye uptake was maximum at a pH of 9.0. The study was carried out in the pH range from 2 to 10 only as zirconium-based MOFs are prone to decomposition in strong alkaline solutions [27].

### 3.6. Adsorption isotherms

**Figure 4:**
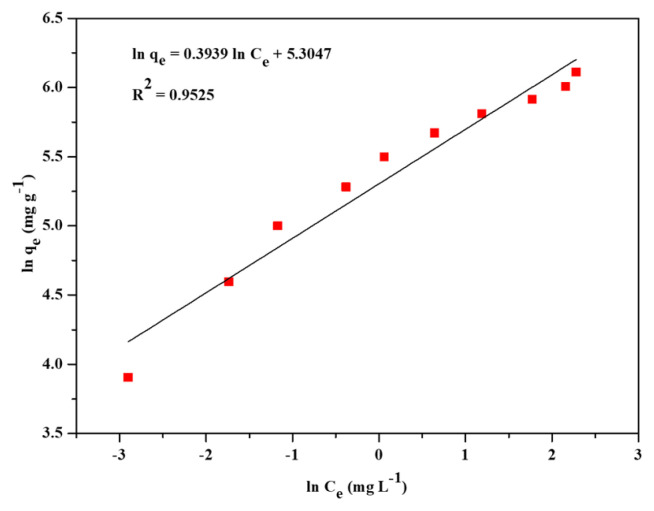
Linear plot for Freundlich adsorption isotherm.

**Figure 5:**
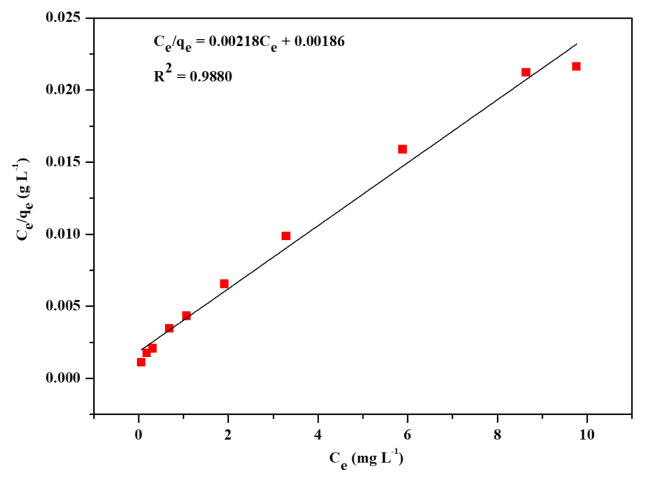
Linear plot for Langmuir adsorption isotherm.

**Figure 6:**
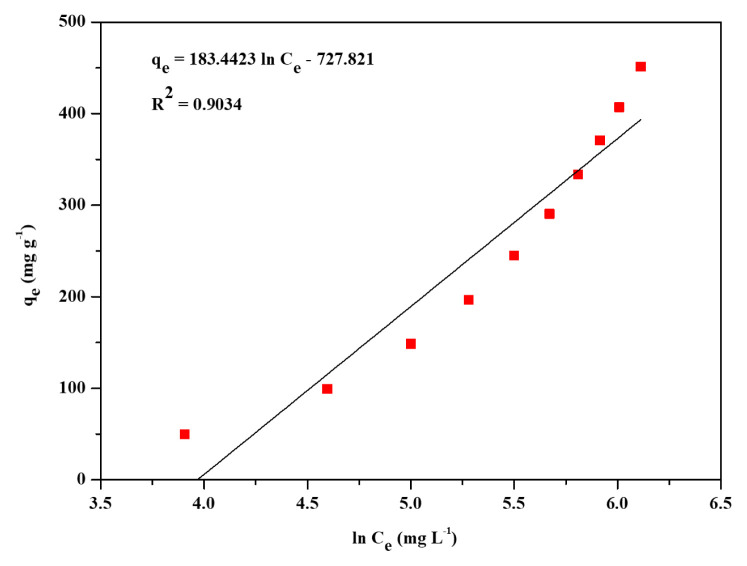
Linear plot for Temkin adsorption isotherm.

The three most widely applied isotherm models, Freundlich, Langmuir, and Temkin, were applied to find out the adsorption performance of Zr-NDC. Adsorption isotherm models were used to provide information about the mechanism of adsorption, evaluation of the performance of the adsorption process, and capacity of Zr-NDC as an adsorbent. These three adsorption isotherms were represented by Equations 3–5. The isotherm data were fitted to the three models to determine the best fit for the adsorption data. Freundlich, Langmuir, and Temkin’s linear plots are shown in Figures 4–6. The adsorption isotherm parameters for the three models are listed in Table 1. Langmuir model (R^2^ = 0.9880) was the best fit compared to Freundlich (R^2^ = 0.9525) and Temkin (R^2^ = 0.9034) models. Hence, the Langmuir adsorption of CV dye from aqueous solution onto Zr-NDC can be best described by the Langmuir adsorption isotherm model. From the slope of the plot in Figure 5, the calculated maximum adsorption capacity (q_m, cal_) of the Zr-NDC adsorbent was found to be 458.71 mg g^−1^. This value was very close to the experimental maximum adsorption capacity (q_m, exp_) value of 454.20 mg g^−1^. From the intercept of the plot, K_L_ was found to be 1.172.

An important characteristic of the Langmuir adsorption isotherm, separation factor (R_L_) was calculated using Equation 10.


Equation 10 $$R_{L}=\frac{1}{1+K_{L}C_{o}}$$

Here C_o_ is the highest initial dye concentration (mg L^−1^) and K_L_ is the Langmuir constant.

With the highest concentration of dye (100 mg L^−1^) used in the adsorption experiment, the calculated value of R_L_ was 0.00846. Since the R_L_ obtained was between 0 and 1 (0 < 0.00846 < 1), the adsorption process was favorable [14]. The results indicate that the Zr-NDC surface was covered by a single layer of CV dye molecules and the active sites were homogeneously distributed on the Zr-NDC adsorbent surface.

The adsorption capacity of Zr-NDC for the adsorption of CV dye was compared with various other MOF adsorbents reported in the literature as shown in Table 2. The maximum CV dye adsorption capacity of Zr-NDC was higher than the adsorption capacity of most of the other MOF adsorbents.

### 3.7. Kinetics studies

**Figure 7:**
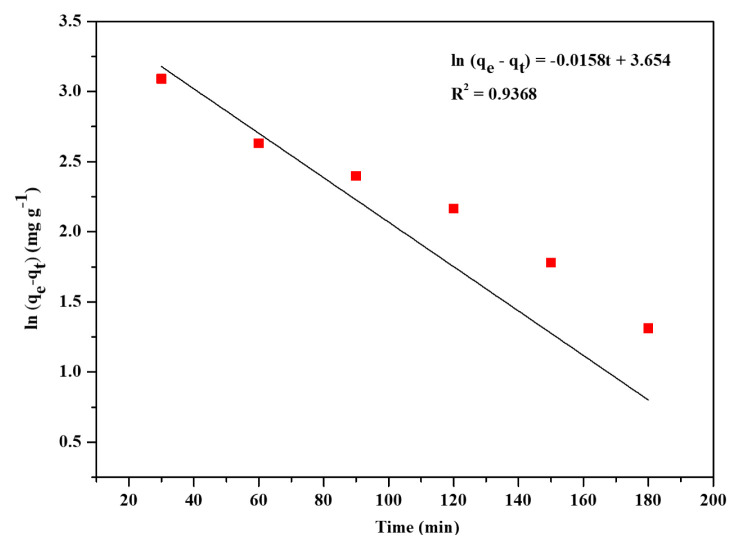
Linear plot of pseudo-first-order kinetics of CV adsorption.

**Figure 8:**
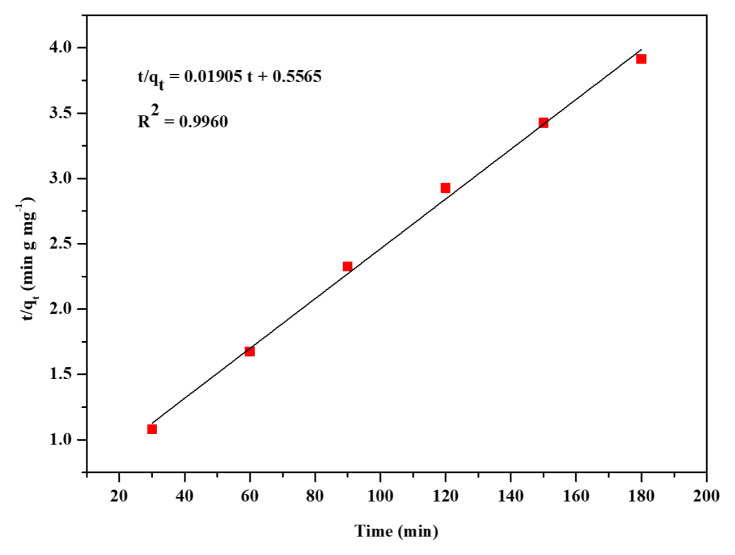
Linear plot of pseudo-second-order kinetics of CV dye adsorption.

**Figure 9:**
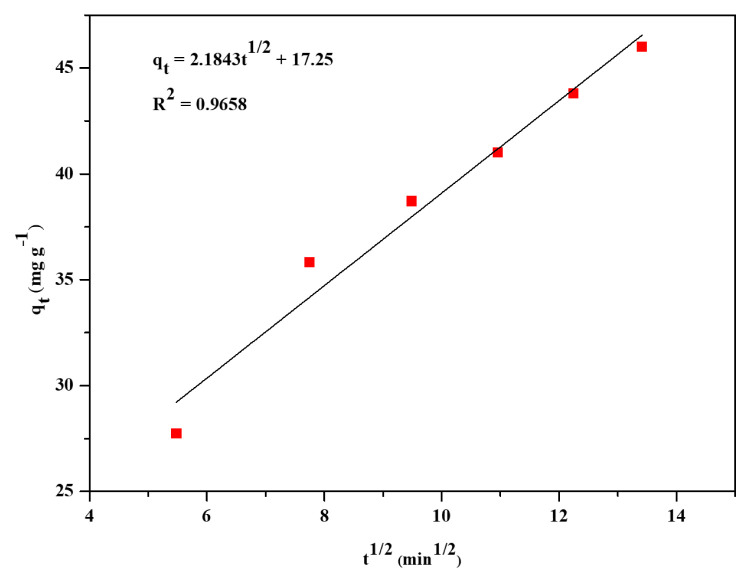
Linear plot of intraparticle diffusion kinetics of CV dye adsorption.

To study the adsorption kinetics, three kinetic models, pseudo-first-order, pseudo-second-order, and intraparticle diffusion were used. These three models were represented by Equations 7–9. The linear plots for the three models were presented in Figures 7–9. The kinetic parameters for the three models are listed in Table 3. Based on the linear regression coefficient value (R^2^), it was found that the pseudo-second-order kinetic model is the best fit for the adsorption kinetics data obtained in the present study. The value of R^2^ (0.9960) for the pseudo-second-order kinetic plot is higher than the R^2^ values of linear plots of the other two kinetics models. The calculated maximum adsorption capacity (q_m, cal_ = 52.49 mg g^−1^) was in good agreement with the experimental value (q_m,_ = 52.49 mg g^−1^). The pseudo-second-order rate constant was determined to be 6.52 × 10^−4^ g mg^−1^ min^−1^. The results obtained in the kinetics studies suggest that the adsorption of CV from aqueous solution onto the Zr-NDC surface proceeds via chemisorption [16].

### 3.8. Adsorption mechanism

Based on the analyses of adsorption isotherm data, it is clear that the Zr-NDC has a good adsorption capacity for CV. Even though a detailed study is necessary to understand the adsorption mechanism, based on the earlier studies, the adsorption may be primarily due to the electrostatic interaction between anionic Zr-NDC and the cationic CV [46]. CV is a cationic dye that usually exists in a positive form. Therefore, any adsorbent having a negative framework will be in electrostatic interaction with CV. With the increase in pH of the solution, there was an increase in the adsorption capacity of the Zr-NDC adsorbent. This indicates that at higher pH values, the Zr-NDC surface carries the negative charge which benefits the adsorption of cationic CV dye through electrostatic interaction. A similar adsorption mechanism via electrostatic interaction has been reported [47]. π-π interactions between planar naphthalene rings in the skeleton of Zr-NDC and benzene rings in the CV may be another possible mechanism to explain the adsorption of CV onto Zr-NDC.

## 4. Conclusions

This study reports the successful synthesis of Zr-NDC for the first time using environmentally friendly ultrasonic-assisted synthesis. The reaction conditions were optimized for the synthesis of Zr-NDC via modulated synthesis approach using 30 equivalents of acetic acid. The removal of CV dye from aqueous solution using pristine Zr-NDC was investigated. A study of adsorption kinetics and adsorption isotherm models revealed that pseudo-second-order kinetics and Langmuir adsorption isotherm model best describe the adsorption of CV dye onto Zr-NDC. The maximum equilibrium adsorption capacity (454.2 mg g^−1^) was achieved with the CV dye having an initial concentration of 100 mg L^−1^. Zr-NDC was found to be a highly efficient adsorbent for the removal of CV dye from aqueous solution.

## Figures and Tables

**Scheme:**
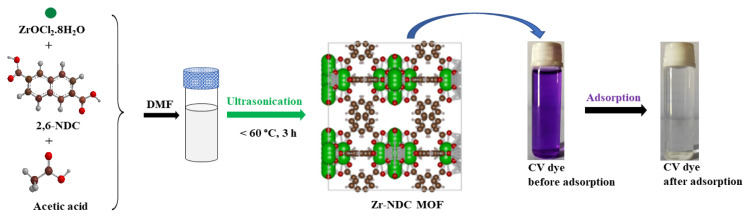
Schematic diagram for the synthesis of Zr-NDC MOF and its adsorption of CV dye from aqueous solution.

**Table 1:** Isotherm parameters for adsorption of crystal violet on Zr-NDC MOF.

Concentration of dye (mg L^−1^)		q_e, exp_ (mg g^−1^)	Freundlich isotherm			Langmuir isotherm			Temkin isotherm		
C_o_	C_e_		n	K_F_ (mg g^−1^)	R^2^	q_m_ (mg g^−1^_)_	K_L_ (L mg^−1^)	R^2^	B_T_ (J)	K_T_ (L mg^−1^)	R^2^
10	0.055	49.725	2.54	201	0.9525	458.71	1.172	0.9880	13.7	0.01926	0.9034
20	0.19	99.05									
30	0.33	148.35									
40	0.73	196.35									
50	1.92	240.4									
60	3.54	282.3									
70	5.69	321.55									
80	7.062	364.69									
90	8.215	408.92									
100	9.16	454.2									

**Table 2:** Comparison with other MOF adsorbents for adsorption of crystal violet dye.

MOF adsorbent	Adsorption capacity (mg g^−1^)	Reference
Zr-NDC	454.2	Present work
Fe-BDC	9.286	[14]
Cu_3_(BTC)_2_	0.29	[15]
H_2_dtoaCu	165.83	[16]
IFMC-2	2.4	[17]
Cd-based	221	[18]
Zn-based	54.50	[19]
BUT-29	832	[20]
Zr-based loaded on PU foam	63.38	[21]
Sr-based phosphotungstic acid	237	[22]
Cu-based	Not reported	[23]

**Table 3:** Kinetic parameters for adsorption of crystal violet on Zr-NDC MOF.

C_i (_mg L^−1^)	q_e, exp_ (mg g^−1^)	Pseudo-first-order kinetic model			Pseudo-second-order kinetic model			Intraparticle diffusion model		
		q_e, cal_ (mg g^−1^)	k_1_ (min^−1^)	R^2^	q_e, cal_ (mg g^−1^)	k_2_ (g mg^−1^ min^−1^)	R^2^	k_i_ (mg g^−1^ min^−1/2^)	C	R^2^
10	49.72	38.61	0.0158	0.9368	52.49	0.0006522	0.9960	2.1843	17.25	0.9658

## References

[1] Rahmat M Rehman A Rahmat S Bhatti HN Iqbal M Highly efficient removal of crystal violet dye from water by MnO2 based nanofibrous mesh/photocatalytic process Journal of Materials Research and Technology 2019 8 5154 5159 10.1016/j.jmrt.2019.08.038

[2] Mittal A Mittal J Malviya A Kaur D Gupta VK Adsorption of hazardous dye crystal violet from wastewater by waste materials Journal of Colloid and Interface Science 2010 343 463 473 10.1016/j.jcis.2009.11.060 20045526

[3] Mani S Bhargava RN Exposure to crystal violet is toxic, genotoxic, and carcinogenic effects on the environment and its degradation and detoxification for environmental safety Reviews of Environmental Contamination and Toxicology 2016 237 71 104 10.1007/978-3-319-23573-8_4 26613989

[4] Dutta S Gupta B Srivastava SK Gupta AK Recent advances on the removal of dyes from wastewater using various adsorbents: a critical review Materials Advances 2021 2 4497 4531 10.1039/d1ma00354b

[5] Zhou Y Jian Lu Yi Zhou Yongdi Liu Recent advances for dyes removal using novel adsorbents: A review Environmental Pollution 2019 252 Part A 352 365 10.1016/j.envpol.2019.05.072 31158664

[6] Katheresan V Kansedo J Lau SY Efficiency of Various Recent Wastewater Dye Removal Methods: A Review Journal of Environmental Chemical Engineering 2018 6 4 4676 4697 10.1016/j.jece.2018.06.060

[7] Bożęcka AM Orlof-Naturalna MM Kopeć M Methods of Dyes Removal from Aqueous Environment Journal of Ecological Engineering 2021 22 9 111 118 10.12911/22998993/141368.

[8] Drout RJ Robinson L Chen Z Islamoglu T Farha OK Zirconium Metal–Organic Frameworks for Organic Pollutant Adsorption Trends in Chemistry 2019 1 3 304 317 10.1016/j.trechm.2019.03.010

[9] Beydaghdari M Saboor FH Babapoor A Karve VV Asgari M Recent Advances in MOF-Based Adsorbents for Dye Removal from the Aquatic Environment Energies 2022 15 2023 10.3390/en15062023

[10] Butova VV Aboraia AM Solayman M Yahia IS Zahran The joint effect of naphthalene-system and defects on dye removal by UiO-66 derivatives Microporous and Mesoporous Materials 2021 325 111314 10.1016/j.micromeso.2021.111314

[11] Fu M Deng X Wang S-Q Yang F Lin L-C Scalable robust nano-porous Zr-based MOF adsorbent with high-capacity for sustainable water purification Separation and Purification Technology 2022 288 120620 10.1016/j.seppur.2022.120620

[12] Abdi J Banisharif F Khataee A Amine-functionalized Zr-MOF/CNTs nanocomposite as an efficient and reusable photocatalyst for removing organic contaminants Journal of Molecular Liquids 2021 334 116129 10.1016/j.molliq.2021.116129

[13] Çiftlik A Semerci TG Şahin O Semerci F Two-Dimensional Metal–Organic Framework Nanostructures Based on 4,4′-Sulfonyldibenzoate for Photocatalytic Degradation of Organic Dyes Crystal Growth & Design 2021 21 3364 3374 10.1021/acs.cgd.1c00152

[14] Soni S Bajpai PK Bharti D Mittal J Arora C Removal of crystal violet from aqueous solution using iron based metal organic framework Desalination Water Treatment 2020 205 386 399 10.5004/dwt.2020.26387

[15] Loera-Serna S Garcia-Ortiz J Ortiz E Dyes Adsorption on Cu3(BTC)2 Metal-Organic Framework Advanced Materials: TechConnect Briefs 2016 1 331 334

[16] Li X Zheng L Huang L Zheng O Lin Z Adsorption Removal of Crystal Violet from Solution Using a Metal-Organic Frameworks Material, Copper Coordination Polymer with Dithiooxamide Journal of Applied Polymer Science 2013 129 2857 2864 10.1002/APP.39009

[17] Qin J-S Zhang S-R Du D-Y Shen P Bao S-J A Microporous Anionic Metal–Organic Framework for Sensing Luminescence of Lanthanide(III) Ions and Selective Absorption of Dyes by Ionic Exchange Chemistry - A European Journal 2014 20 5625 5630 10.1002/chem.201304480 24677301

[18] Chand S Elahi SM Pal A Das MC A new set of Cd(II)-coordination polymers with mixed ligand of dicarboxylate and pyridyl substituted diaminotriazine: selective sorption towards CO2 and cationic dye Dalton Transactions 2017 46 9901 9911 10.1039/C7DT01657C 28722040

[19] Zhang J Li F Sun Q Rapid and selective adsorption of cationic dyes by a unique metal-organic framework with decorated pore surface Applied Surface Science 2018 440 1219 1226 j.apsusc.2018.01.258

[20] Yang Q Wang B Chen Y Xie Y An anionic In(III)-based metal-organic framework with Lewis basic sites for the selective adsorption and separation of organic cationic dyes Chinese Chemical Letters 2019 30 234 238 10.1016/j.cclet.2018.03.023

[21] Zhao J Xu L Su Y Yu H Liu H Zr-MOFs loaded on polyurethane foam by polydopamine for enhanced dye adsorption Journal of Environmental Sciences 2021 101 177 188 10.1016/j.jes.2020.08.021 33334514

[22] Ibrahim AA Ali SL Adly MS El-Hakam SA Samra SE Ahmed AL Green construction of eco-friendly phosphotungstic acid Sr-MOF catalysts for crystal violet removal and synthesis of coumarin and xanthene compounds RSC Advances 2021 11 37276 37299 10.1039/D1RA07160B 35496434 PMC9043797

[23] Abbasi AR Karimi M Daasbjerg K Efficient removal of crystal violet and methylene blue from wastewater by ultrasound nanoparticles Cu-MOF in comparison with mechanosynthesis method Ultrasonics Sonochemistry 2017 37 182 191 10.1016/j.ultsonch.2017.01.007 28427622

[24] Sun Z Wu X Qu K Huang Z Liu S Bimetallic metal-organic frameworks anchored corncob-derived porous carbon photocatalysts for synergistic degradation of organic pollutants Chemosphere 2020 259 127389 10.1016/j.chemosphere.2020.127389 32590175

[25] Howarth AJ Liu Y Li P Li Z Wang TC Chemical, thermal and mechanical stabilities of metal-organic frameworks Nature Reviews Materials 2016 1 15018 10.1038/natrevmats.2015.18

[26] Chen Z Hanna SL Redfern LR Alezi D Islamoglu T Reticular chemistry in the rational synthesis of functional zirconium cluster-based MOFs Coordination Chemistry Reviews 2019 386 32 49 10.1016/j.ccr.2019.01.017

[27] Bai Y Dou Y Xie L-H Rutledge W LJ-R Zr-based metal-organic frameworks: design, synthesis, structure, and applications Chemical Society Reviews 2016 45 6 2327 2367 10.1039/C5CS00837A 26886869

[28] Schaate A Roy P Godt A Lippke J Waltz F Modulated Synthesis of Zr-based Metal-Organic Frameworks: From Nano to Single Crystals Chemistry-A European Journal 2011 17 24 6643 6651 10.1002/chem.201003211 21547962

[29] Safarifard V Morsali A Applications of ultrasound to the synthesis of nanoscale metal-organic coordination polymers Coordination Chemistry Reviews 2015 292 1 14 10.1016/j.ccr.2015.02.014

[30] Haque E Khan NA Park JH Jhung SH Synthesis of a Metal-Organic Framework Material, Iron Terephthalate, by Ultrasound, Microwave, and Conventional Electric Heating: A Kinetic Study Chemistry-A European Journal 2010 16 3 1046 1052 10.1002/chem.200902382 20014080

[31] Zhang W Huang H Liu D Yang Q Xiao Y A new metal-organic framework with high stability based on zirconium for sensing small molecules Microporous Mesoporous Materials 2013 171 118 124 10.1016/j.micromeso.2013.01.003

[32] Bon V Senkovska I Weiss MS Kaskel S Tailoring of network dimensionality and porosity adjustments in Zr- and Hf-based MOFs CrystEngComm 2013 15 45 9572 9577 10.1039/C3CE41121D

[33] Guillerm V Ragon M Dan-Hardi T Devic T Visnuvarthan M A series of Isoreticular, Highly Stable, Porous Zirconium Oxide Based Metal-Organic Frameworks Angewandte Chemie International Edition 2012 51 37 9267 9271 10.1002/anie.201204806 22887718

[34] David J Trolliard G Volkringer C Loiseau T Maître A Synthesis of zirconium oxycarbide powders using metal-organic framework (MOF) compounds as precursors RSC Advances 2015 5 64 51650 51661 10.1039/C5RA01172H

[35] Gutiérrez M Martin C Kennes K Hofkens J Auweraer MV New OLEDs Based on Zirconium Metal-Organic Framework Advanced Optical Materials 2018 6 6 1701060 10.1002/adom.201701060

[36] Valenzuela L Amariei G Ezugwu CI Faraldos M Bahamonde A Zirconium-based Metal-Organic Frameworks for highly efficient solar light-driven photoelectrocatalytic disinfection Separation and Purification Technology 2022 285 3 120351 10.1016/j.seppur.2021.120351

[37] Saidi M Benomara A Mokhtari M Boukli-Hacene L Sonochemical synthesis of Zr-fumaric based metal-organic framework (MOF) and its performance evaluation in methyl violet 2B decolorization by photocatalysis Reaction Kinetics, Mechanisms and Catalysis 2020 131 2 1009 1021 10.1007/s11144-020-01897-3

[38] Caicedo O Devia-Ramirez J Malagón A Adsorption of Common Laboratory Dyes Using Natural Fibers from Luffa cylindrica Journal of Chemical Education 2018 95 12 2233 2237 10.1021/acs.jchemed.8b00156

[39] Panda J Sahu NS Sahoo JK Biswal SP Pattanayak SK Samantray R Efficient removal of two anionic dyes by a highly robust zirconium based metal organic framework from aqueous medium: Experimental findings with molecular docking study Environmental Nanotechnology, Monitoring and Management 2020 14 100340 10.1016/j.enmm.2020.100340

[40] Wu S-C You X Yang C Cheng J-H Adsorption behaviour of methyl orange onto an aluminum-based metal organic framework, MIL-68(Al) Water Science & Technology 2017 75 12 2800 2810 10.2166/wst.2017.154 28659520

[41] Ardila-Suárez C Rodríguez-Pereira J Baldovino-Medrano VG Ramírez-Caballero GE An analysis of the effect of zirconium precursors of MOF-808 on its thermal stability, and structural and surface properties CrystEngComm 2019 21 9 1407 1415 10.1039/C8CE01722K

[42] Łyszczek R Lipke A Microwave-assisted synthesis of lanthanide 2,6-naphthalenedicarboxylates: Thermal, luminescent and sorption characterization Microporous Mesoporous Materials 2013 168 81 91 10.1016/j.micromeso.2012.09.016

[43] Black CA Costa JS Fu WT Massera C Roubeau O 3-D Lanthanide Metal-Organic Frameworks: Structure, Photoluminescence, and Magnetism Inorganic Chemistry 2009 48 3 1062 1068 10.1021/ic8017826 19128001

[44] Hirschle P Preiß T Auras F Pick A Völkner J Exploration of MOF nanoparticle sizes using various physical characterization methods - is what you measure what you get CrystEngComm 2016 18 23 4359 4368 10.1039/C6CE00198J

[45] Yagub MT Sen TK Afroze S Ang HM Dye and its removal from aqueous solution by adsorption: A review Advances in Colloid and Interface Science 2014 209 172 184 10.1016/j.cis.2014.04.002 24780401

[46] Khan MS Khalid M Shahid M What triggers dye adsorption by metal organic frameworks? The current perspectives Materials Advances 2020 1 1575 1601 10.1039/d0ma00291g

[47] Haque E Jun JW Jhung SH Adsorptive removal of methyl orange and methylene blue from aqueous solution with a metal-organic material, iron terephthalate (MOF-235) Journal of Hazardous Materials 2011 185 507 511 10.1016/j.jhazmat.2010.09.035 20933323

